# Motif depletion in bacteriophages infecting hosts with CRISPR systems

**DOI:** 10.1186/1471-2164-15-663

**Published:** 2014-08-08

**Authors:** Anne Kupczok, Jonathan P Bollback

**Affiliations:** IST Austria, Am Campus 1, 3400 Klosterneuburg, Austria; Institute of Microbiology, Christian-Albrechts-University of Kiel, 24118 Kiel, Germany

**Keywords:** Bacterial immunity, Bacteria-phage coevolution, Selection, PAM

## Abstract

**Background:**

CRISPR is a microbial immune system likely to be involved in host-parasite coevolution. It functions using target sequences encoded by the bacterial genome, which interfere with invading nucleic acids using a homology-dependent system. The system also requires protospacer associated motifs (PAMs), short motifs close to the target sequence that are required for interference in CRISPR types I and II. Here, we investigate whether PAMs are depleted in phage genomes due to selection pressure to escape recognition.

**Results:**

To this end, we analyzed two data sets. Phages infecting all bacterial hosts were analyzed first, followed by a detailed analysis of phages infecting the genus *Streptococcus*, where PAMs are best understood. We use two different measures of motif underrepresentation that control for codon bias and the frequency of submotifs. We compare phages infecting species with a particular CRISPR type to those infecting species without that type. Since only known PAMs were investigated, the analysis is restricted to CRISPR types I-C and I-E and in *Streptococcus* to types I-C and II. We found evidence for PAM depletion in *Streptococcus* phages infecting hosts with CRISPR type I-C, in *Vibrio* phages infecting hosts with CRISPR type I-E and in *Streptococcus thermopilus* phages infecting hosts with type II-A, known as CRISPR3.

**Conclusions:**

The observed motif depletion in phages with hosts having CRISPR can be attributed to selection rather than to mutational bias, as mutational bias should affect the phages of all hosts. This observation implies that the CRISPR system has been efficient in the groups discussed here.

**Electronic supplementary material:**

The online version of this article (doi:10.1186/1471-2164-15-663) contains supplementary material, which is available to authorized users.

## Background

Bacteria harbor diverse defense systems against phages, plasmids, and mobile elements, likely driven by the coevolutionary dynamics between bacteria and their parasites. The recently discovered microbial immune system CRISPR/Cas contains the CRISPR (clustered regularly interspaced short palindromic repeats) locus, an array of sequence-specific repeats flanking unique spacers, and adjacent *cas* (CRISPR associated) genes [1]. Cas genes characteristically show high rates of evolution, both in the protein sequence and in the operon structure resulting in different CRISPR/Cas types [2]. This high diversity has been attributed to host-parasite coevolution [3].

CRISPR mediated immunity acts in three stages. First, new spacer sequences are acquired from invasive elements that are incorporated into the CRISPR array. These sequences are the basis of the sequence-specific immune response. Second, the CRISPR locus is transcribed and processed into mature crRNAs by the associated Cas proteins in a process known as biogenesis. Lastly, in the interference stage, crRNAs and associated Cas proteins target and bind protospacers (sequences complementary to spacers) leading to cleavage and degradation of the foreign genetic material.

PAMs (protospacer associated motifs) are short motif sequences in the nucleotide sequences of the invasive elements recognized by the CRISPR/Cas system. They are necessary for the successful acquisition of a new spacer and for the interference with foreign DNA. PAMs are specific to the different CRISPR types and repeat sequences [4]. Note that the PAM sequences for the acquisition and interference stages may differ slightly [5]. PAM sequences have been identified with different methods, using phage challenge and plasmid elimination experiments, interference experiments, or computational methods (Table 1). The most accurate identification comes from phage challenge and plasmid elimination experiments. In these experiments, new spacers are acquired that are homologous to the protospacer located on the phage or plasmid. PAMs are identified as the conserved motifs occurring next to these protospacers. The reason this method is accurate is that mutations have not yet occurred in the PAM sequence, and it allows the determination of the acquisition motif. In interference experiments, in contrast, PAMs are tested for their ability to interfere with foreign genetic material: different motifs are tested for their ability to be recognized by the CRISPR system, with PAMs identified by their proximity to the recognized motifs. PAMs can be inferred computationally as motifs that are present close to inferred protospacers [4]. This third approach is limited because mutations may have occurred in the PAMs since the original acqusition of the protospacer and hence acquisition and interference motifs cannot be distinguished (e.g., for *S. mutans*, the PAM WAAR was found using computational methods and YAAAWY in phage challenge experiments [6]).

**Table 1:** **Published evidence for PAMs in CRISPR types I and II**

Type	Motif	Species	Evidence	Publication
I-A	GG	*Metallosphaera sedula*,	computational	[4]
		*Sulfolobus solfataricus*		
I-A	GG	*Sulfolobus solfataricus*	interference	[7]
I-A	GG	*Sulfolobus islandicus*	interference	[8]
I-B	ACT,TAA,	*Haloferax volcanii*	interference	[9]
	TAT, TAG,			
	TTC, CAC			
I-B	GG	*Methanothermobacter*,	computational	[4]
		*thermautotrophicus*,		
		*Listeria monocytogenes*		
I-C	GAA	*Streptococcus mutans*	computational	[6]
I-C	GAA	*Streptococcus pyogenes*,	computational	[4]
		*Xanthomonas oryzae*		
I-D	GTY	*Microcystis aeruginosa*	computational	[10]
I-E	AAG	*Erwinia amylovora*	computational	[11]
I-E	AAG	*Pseudomonas aeruginosa*	computational	[4],
				[12]
I-E	AWG	*Escherichia coli*	computational	[4]
I-E	ATG	*Escherichia coli*	interference	[13]
I-E	AAG	*Escherichia coli*	plasmid elimination	[14],
				[15]
I-E	AAG,ATG,	*Escherichia coli*	interference	[16]
	AGG,GAG			
I-E	AAY	*Gardnerella vaginalis*	computational	[17]
I-E	AAY	*Lactobacillus casei*	computational	[18]
I-F	GG	*Escherichia coli*	interference	[19]
I-F	GG	*Pectobacterium atrosepticum*	interference	[20]
I-F	GG	*Pseudomonas aeruginosa*	computational	[4],
				[12]
I-F	GG	*Shewanella spp.*	computational	[4]
II	GG	*Streptococcus agalactiae*,	computational	[4]
		*Streptococcus pyogenes*,		
		*Listeria monocytogenes*		
II	GG	*Streptococcus agalactiae*	interference	[21]
II	GG or	*Streptococcus mutans*	computational	[6]
	WAAR			
II	YAAAWY	*Streptococcus mutans*	phage challenge	[6]
II	GGNG	*Streptococcus thermophilus*	computational	[22]
II	GGNG	*Streptococcus thermophilus* ^∗^	interference	[23]
II	GG	Streptococcus pyogenes,	interference	[24]
		*Streptococcus mutans*,		
		*Streptococcus thermophilus*,		
		*Francisella novicida*		
II	ACA	*Campylobacter jejuni*	interference	[24]
II	GATT	*Neisseria meningitidis*	interference	[24]
II	GNNNCNNA	*Pasteurella multocida*	interference	[24]
II	AGAAW	*Streptococcus thermophilus*	phage challenge	[25]
II	AAAAW	*Streptococcus thermophilus*	interference	[24]
II	TGAAA	*Lactobacillus casei*	computational	[18]

The PAM orientation is displayed arbitrarily. See text for details on the different types of evidence. ^*^ - the interference function of the system was shown in *E. coli*.

Given the importance of these motifs for both spacer acquisition and interference, we hypothesize that PAM sequences will be selected against if they occur in the genomes of phages co-evolving with CRISPR containing hosts. Selection against PAMs might act in two ways. First, mutations disrupting PAM sequences may allow phages to avoid CRISPR recognition in the interference stage [25]. Second, a dearth of these sequences in the genome may allow phages to escape part of their genomes being acquired as a protospacer by the CRISPR system in the first place. We test this hypothesis for well-defined PAM sequences used by CRISPR types I and II (Table 1), focusing on well-understood subtypes of these groups.

We investigate the underrepresentation of PAMs in bacteriophages that encounter CRISPR systems. Specifically, we test for a depletion of PAMs in phages associated with host species harboring CRISPR/Cas (denoted as CRISPR ^+^) by comparing them to phages associated with host species of the same genus not harboring CRISPR/Cas (CRISPR ^-^). This allows us to detect selection acting against PAMs, even if the effect is weaker than for other factors affecting genome composition, such as codon usage, correct location of transcription factor binding sites, or mutational biases, as these forces should be uncorrelated with the presence of CRISPR in the host.

We employ two complementary measures of motif underrepresentation, or depletion, that account for mutational bias and selection in different ways (see also Material and Methods). The *resampling method* controls for codon bias [26] and accounts for selection on amino acid content and codon usage. The *substring method* controls for the frequency of substrings of length *n* - 1 of a motif of length *n*[27] and accounts for selection pressure and mutational bias on submotifs of length *n* - 1. We use both methods to control for mutational bias and sources of selection other than immune avoidance. Both methods result in a ratio of observed-over-expected frequencies, and the log2 ratio of the PAM is denoted as *r*_PAM_. *r*_PAM_ is an indication of over- (*r*_PAM_>0) or underrepresentation (*r*_PAM_<0) of the PAM in a particular phage genome. For more powerful analyses in the presence of other selective factors, *r*_PAM_-values of CRISPR ^+^ are compared to CRISPR ^-^ using the Wilcoxon rank-sum test. *r*_PAM_-values that are significantly smaller in CRISPR ^+^ are an indication of PAM depletion due to selection to avoid the CRISPR defense system.

We present the results for all phage genomes with annotated hosts. To match phages and hosts, we made use of the /host annotation from the genbank file. Notably this generally contains the information of the bacteria the phage was isolated from and not the full host range. In the second part we present more detailed results for the genus *Streptococcus*. Several facts make the genus *Streptococcus* a good model system to study this question. First, the function of the CRISPR system was first described in *S. thermophilus*[28], and active CRISPR systems were also reported in other *Streptococcus* species [6, 21]. Second, different CRISPR systems are present in different species (Additional file 1: Tables S1,S2), and the PAM sequences have been studied for the different systems. Third, phages have been described and sequenced for this genus, and their host specificity is known (Additional file 1: Table S3).

## Results

### Phages infecting all bacteria

We analyzed two data sets, one including any suitable data, and one focusing on the *Streptococcus* genus, where the CRISPR system is particualarly well understood. For the first data set, we analyzed available sequence from phages infecting known and sequenced bacterial species (i.e., with the /host-tag set in the NCBI database), comprising 688 genomes for phages infecting 129 different bacterial hosts (Additional file 2).

#### CRISPR type I-C

**Figure 1:**
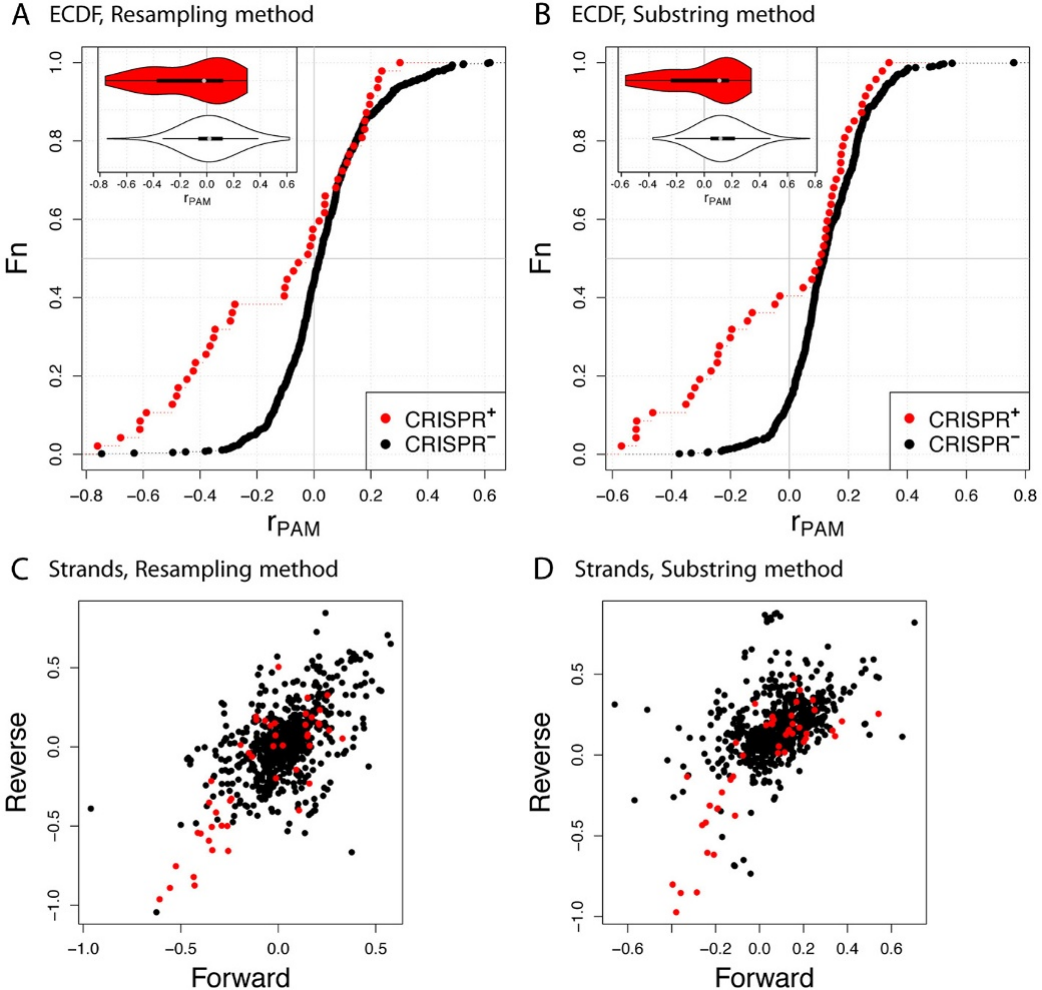
**Distributions of** ***r***_**PAM**_ **for type I-C, PAM GAA and two different measures of underrepresentation.** **A**, **B**: Combined ratio for GAA (motif GAA or TTC). Main plots - empirical cumulative density functions (ECDF), Fn - cumulative probability, i.e., the frequency of observations having that *r*_PAM_ value or a smaller value; gray horizontal line indicates median; inserted plots - distribution as a violin plot [29]. In all plots, the gray vertical line marks *r*_PAM_=0; i.e., the observed frequency equals the expected frequency. **C**, **D**: Separate ratios for forward strand (motif GAA) and reverse strand (motif TTC).

CRISPR type I-C has the PAM GAA for all the species where the PAM was studied (Table 1). We found that both methods, resampling and substring, resulted in smaller *r*_PAM_-values for phages infecting bacterial hosts with CRISPR (CRISPR ^+^) compared to those infecting hosts without CRISPR (CRISPR ^-^) when using all phages infecting bacteria (Figure 1A,B). Computing ratios separately for the forward and reverse strand results in a high correlation of the respective ratios (Pearson’s correlation coefficient 0.58 for the resampling method and 0.49 for the substring method) with a stronger depletion of the PAM on the reverse strand (Figure 1C,D). Because of this strong correlation we combine ratios for both strands, unless stated otherwise. Using both methods, the difference between CRISPR ^+^ and CRISPR ^-^ phages is significant using the Wilcoxon rank-sum test (line “Bacteria”, Table 2). These results might be affected by a few host species with data for a large number of phages; however resampling the data set to give an even host distribution still yields significant results in most cases (Table 2).

**Table 2:** **Summary of the statistical results using the Wilcoxon rank-sum test for CRISPR type I-C and PAM GAA**

***r***_PAM_					
CRISPR ^-^	CRISPR ^+^	Difference	***p*** -value	Strain resampling	Method
Bacteria: 12 (47) CRISPR^+^, 117 (641) CRISPR^-^					
0.01883	-0.02136	0.04019	0.007858	99	res
0.1169	0.1082	0.008770	0.009564	99	
*Bacillus*: 2 (18) CRISPR^+^, 4 (18) CRISPR^-^					
0.09768	0.1080	-0.01033	0.1916	-	res
0.08673	0.1773	-0.09062	0.0004285	100	sub
*Lactobacillus*: 1 (3) CRISPR^+^, 6 (13) CRISPR^-^					
-0.02019	-0.01306	-0.007136	0.6107	-	res
0.005076	-0.04924	0.05421	0.5214	-	sub
*Streptococcus*: 3 (9) CRISPR^+^, 7 (22) CRISPR^-^					
-0.04927	-0.5889	0.5397	4.464e-06	100	res
-0.03720	-0.4635	0.4263	1.19e-0.06	100	sub
*Streptococcus* (extended)					
-0.06292	-0.3772	0.3143	1.965e-05	99	res
-0.04374	-0.3208	0.2771	1.223e-05	99	sub

For each data set, the number of species (strains) in CRISPR^+^ and CRISPR^-^, respectively, is given. Median of *r*_PAM_ (log-ratio of the PAM) is given for CRISPR^+^ and CRISPR^-^. “Difference” is the difference of these values, positive values indicate underrepresentation of the PAM in CRISPR^+^. Column “Strain resampling”: for significant results, resampling to a uniform species distribution, number of significant (*p* < 0.05) outcomes out of 100. Column “Method”: “res” - resampling method, “sub” - substring method.

To see whether our results also hold for closely related hosts, we repeated the analysis based on bacterial genera with at least three CRISPR ^-^ and three CRISPR ^+^ phages. For type I-C, this condition is only fulfilled for the genera *Bacillus*, *Lactobacillus* and *Streptococcus*. Of these, we only found evidence for PAM depletion in CRISPR ^+^ for *Streptococcus* phages (see Table 2 and the section on *Streptococcus*).

#### CRISPR type I-E

Several PAMs have been described for type I-E. For Gammaproteobacteria, the acquisition motif AAG and the interference motif AWG are known, while, for an Actinobacteria and a Firmicute, the motif AAY was found.

**Figure 2:**
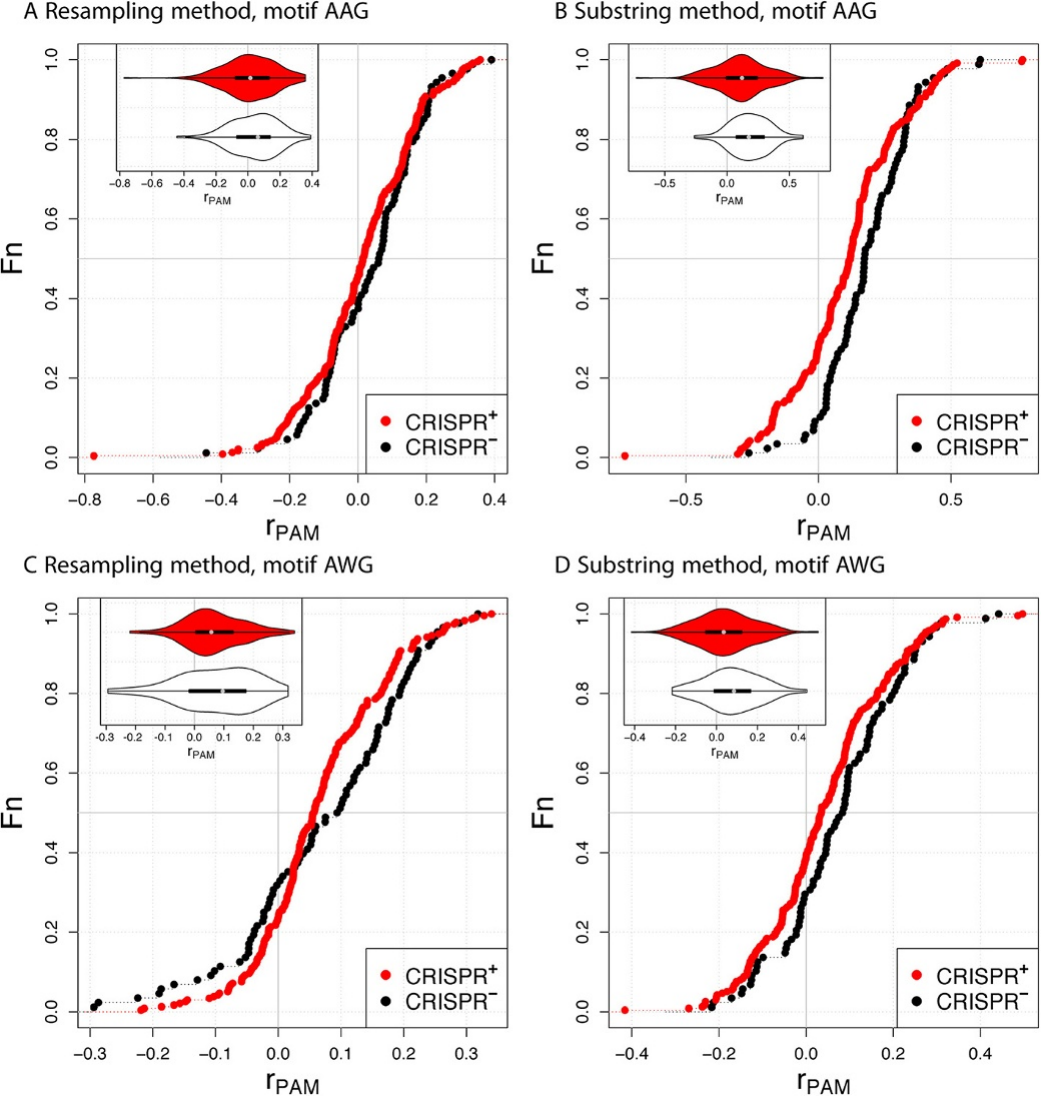
**Distributions of** ***r***_**PAM**_ **for type I-E and two different measures of underrepresentation.** Only phages with host Gammaproteobacteria are shown. See also legend in Figure 1.

**Figure 3:**
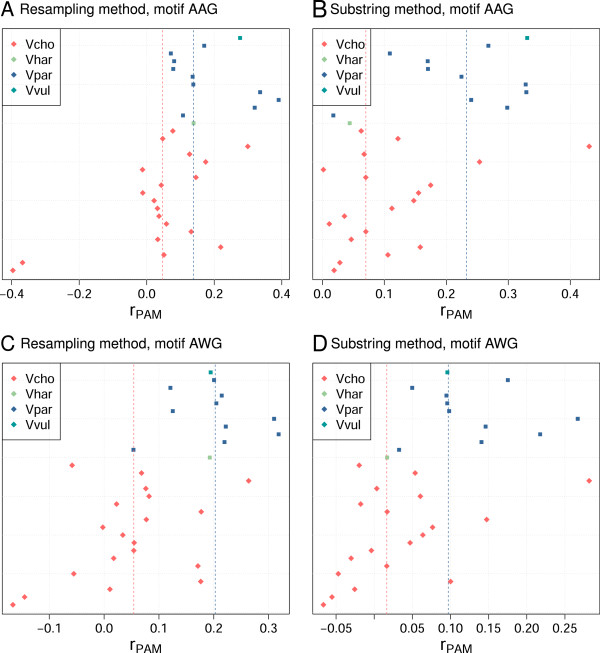
***r***_**PAM**_ **-values for type I-E for genus** ***Vibrio*** **.** Each line shows one phage genome and is colored according to host species. Median of *r*_PAM_ for CRISPR ^+^ (Vcho) is given as a red line and for CRISPR ^-^ (Vhar, Vpar and Vvul) as a blue line. Abbreviations: Vcho - *Vibrio cholerae*, Vhar - *Vibrio harveyi*, Vpar - *Vibrio parahaemolyticus*, Vvul - *Vibrio vulnificus*; strains within a host species are ordered arbitrarily as follows (bottom to top). *Vibrio cholerae*: CP-T1, vB_VchM-138, fs2, JA-1, VFJ, K139, VSK, fs1, VGJphi, VP2, VP5, KSF-1phi, kappa, VEJphi, ICP1, ICP2, ICP3, CTX, VCY-phi. *Vibrio harveyi*: VHML. *Vibrio parahaemolyticus*: pVp-1, VfO3K6, VPMS1, VfO4K68, VpV262, KVP40, Vf33, Vf12, VP882, VP93. *Vibrio vulnificus*: VvAW1.

In Gammaproteobacteria, the ratios for both AAG and AWG are, on average, larger than zero, indicating overrepresentation (Table 3, Figure 2). CRISPR ^+^ phages show generally smaller ratios compared to CRISPR ^-^, but the difference is significant only with the substring method. However, the result for AWG could have been biased by the species distribution. To find particular hosts for which phages show a depletion, we repeated the analysis for the two Gammaproteobacteria genera with at least three CRISPR ^-^ and three CRISPR ^+^ phages, *Pseudomonas* and *Vibrio*. Of these, *Vibrio* shows a clear depletion of the patterns AAG and AWG in CRISPR ^+^ (Table 3, Figure 3).

**Table 3:** **Summary of the statistical results using the Wilcoxon rank-sum test for CRISPR type I-E**

	***r***_PAM_					
PAM	CRISPR ^-^	CRISPR ^+^	Difference	***p*** -value	Strain resampling	Method
Bacteria: 28 (297) CRISPR^+^, 101 (391) CRISPR^-^						
AAG	0.7174	-0.001687	0.07342	4.499e-07	92	res
AAG	0.1177	0.1201	-0.002424	0.03667	32	sub
AWG	0.5161	0.04535	0.006267	0.6928	-	res
AWG	0.05169	0.02959	0.02209	0.001556	66	sub
AAY	-0.009426	0.02582	-0.03525	1.461e-06	100	res
AAY	-0.03633	-0.01978	-0.01656	7.319e-06	99	sub
Gammaproteobacteria: 17 (239) CRISPR^+^, 27 (88) CRISPR^-^						
AAG	0.06205	0.01468	0.04737	0.1733	-	res
AAG	0.1745	0.1201	0.05443	0.0009832	99	sub
AWG	0.09614	0.05743	0.03871	0.3779	-	res
AWG	0.08496	0.03524	0.04972	0.03247	49	sub
*Pseudomonas*: 1 (54) CRISPR^+^, 4 (23) CRISPR^-^						
AAG	0.09970	09930	0.0004042	0.8282	-	res
AAG	0.2794	0.2654	0.01402	0.4867	-	sub
AWG	0.05831	0.1013	-0.04294	0.4525	-	res
AWG	0.1649	0.1636	0.001262	0.2403	-	sub
*Vibrio*: 1 (19) CRISPR^+^, 3 (12) CRISPR^-^						
AAG	0.1384	0.04731	0.09112	0.002313	99	res
AAG	0.2323	0.07019	0.1621	0.01414	81	sub
AWG	0.2026	0.05405	0.1485	0.0001185	99	res
AWG	0.09707	0.01647	0.08060	0.003148	99	sub
not Proteobacteria: 10 (49) CRISPR^+^, 54 (243) CRISPR^-^						
AAY	-0.01711	0.007357	-0.009751	0.2059	-	res
AAY	-0.2643	-0.04995	0.02352	0.003247	68	sub
*Lactobacillus*: 2 (5) CRISPR^+^, 5 (11) CRISPR^-^						
AAY	-0.02047	-0.02471	-0.005757	1	-	res
AAY	0.01788	-0.029311	0.05710	0.06868	-	sub
*Mycobacterium*: 1 (12) CRISPR^+^, 2 (3) CRISPR^-^						
AAY	0.006024	0.1491	-0.1431	0.9451	-	res
AAY	-0.06072	0.0009736	-0.06170	0.3648	-	sub

See also caption in Table 2.

**Figure 4:**
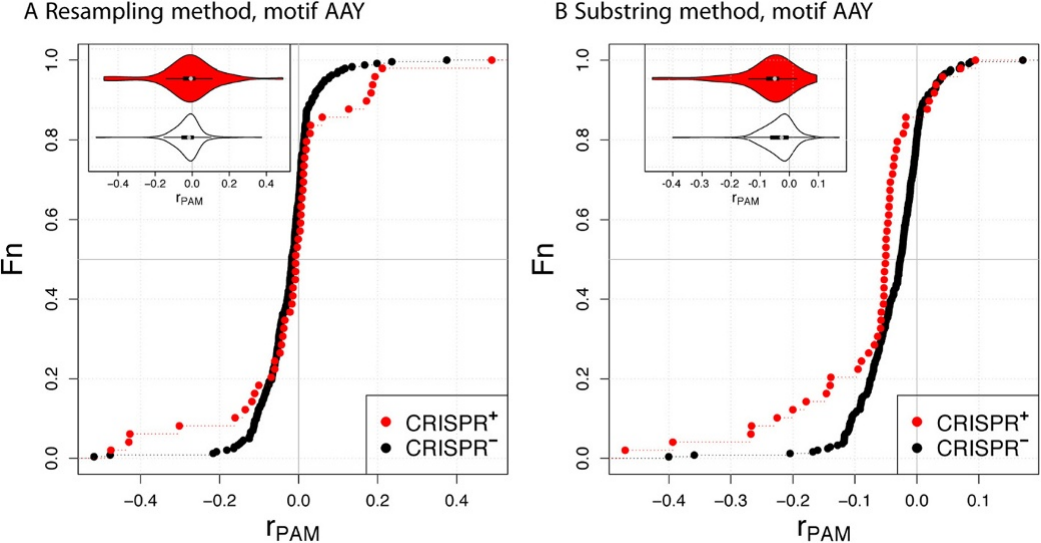
**Distributions of** ***r***_**PAM**_ **for type I-E and two different measures of underrepresentation.** Only phages for hosts other than Proteobacteria are shown. See also legend in Figure 1.

Next, we analyzed the motif AAY observed or the Actinobacteria and a Firmicute. When we consider all hosts, including non-Actinobacteria and non-Firmicute, the motif AAY is overrepresented in CRISPR ^+^ (Table 3, Figure 4). Note that the majority of these hosts are Gammaproteobacteria, whose phages show depletion of the motif AAG. It may be that the AAG depletion and the AAY overrepresenation are related, as AAG motifs can be eliminated by a single mutation to AAY. Phages infecting hosts other than Proteobacteria show a depletion in AAY only with the substring method. The only non-Proteobacteria genera with at least three CRISPR ^+^ and three CRISPR ^-^ phages are *Lactobacillus* and *Mycobacterium*, both of which show no evidence of PAM depletion (Table 3).

### Phages infecting *Streptococcus*

We next focus on the genus *Streptococcus*. We extended this data set by including phages not in the genome database but in the nucleotide database and by including phages without a /host tag. This resulted in 44 phages (Additional file 1: Table S3). We group phages based on the host species, which is well-founded here as species in this genus are found to be monophyletic (Additional file 1: Figure S1).

Different types of CRISPR types I and II were present and distributed over the *Streptococcus* phylogeny (Additional file 1: Table S1). Type I-F was only present in one species and was ignored in the following analysis. For type I-E, the motif was not known precisely (see previous section), so we also did not consider that type. Two kinds of type II-A were present that could be distinguished through different *csn2* homologs and different repeats. All types show the presence of PAMs (Additional file 1: Figure S2, displayed using WebLogo [30]). *r*_PAM_ values for the motifs analyzed here are given in Additional file 3.

#### CRISPR type I-C

**Figure 5:**
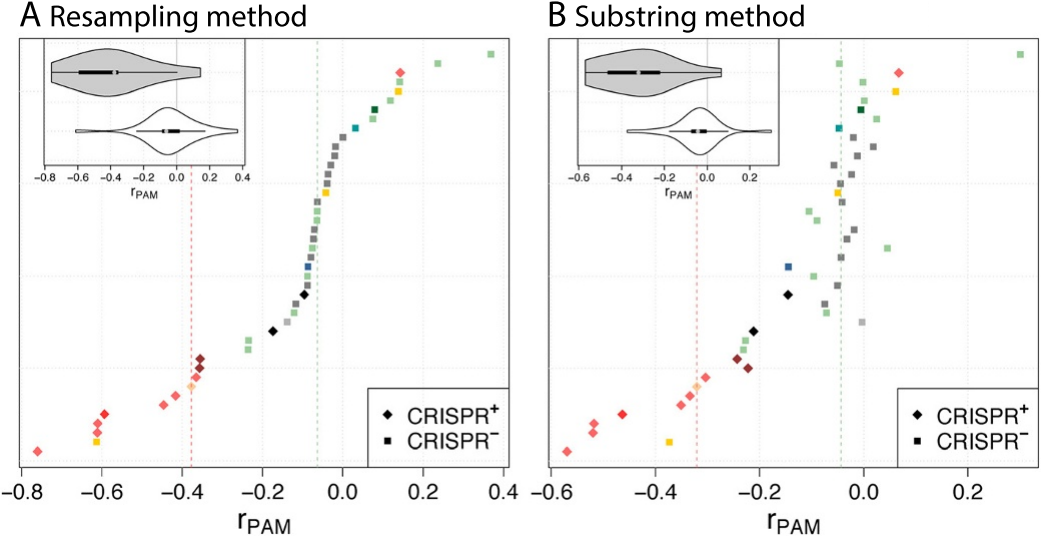
***r***_**PAM**_ **-values for type I-C, PAM GAA, and genus** ***Streptococcus*** **.** Main plot: Each line shows one phage genome and is colored according to host species (Additional file 1: Figure S1). Median of *r*_PAM_ for CRISPR ^+^ is given as a red line and for CRISPR ^-^ as a green line. Inserted plot: Distributions of *r*_PAM_ for CRISPR ^+^ (gray) and CRISPR ^-^ (white). Strains are ordered by *r*_PAM_ of the resampling method (bottom to top): 315.5, phiNJ2, 315.4, 315.1, phi3396, 315.2, 315.3, P9, 315.6, LYGO9, JX01, MM1, MM1_1998, M102AD, YMC-2011, 2167, Abc2, M102, Sfi21, 34117, PH15, DT1, Cp-1, O1205, 2972, V22, 8140, Sfi11, SMP, ALQ13.2, TP-J34, Sfi19, 7201, 858, 5093, SM1, EJ-1, PH10, 11865, phi-SsUD.1, 23782, phi-m46.1, 040922, Dp-1.

As a preliminary step, we identified the PAM for CRISPR type I-C bioinformatically by inferring consensus motifs adjacent to inferred protospacers. We recover the previously reported PAM GAA for CRISPR type I-C [31]. We found this motif to be underrepresented in CRISPR ^+^ phages (diamonds in Figure 5) compared to CRISPR ^-^ phages (squares in Figure 5). This difference is robust to whether the resampling method or the substring method is used (Table 2). A similar pattern was observed when the prophages were analyzed (Additional file 1: Figure S3).

There may, however, be an issue of phylogenetic non-independence. Hosts with CRISPR are all from the pyogenic group or *S. mutans*, and their phages could be related as well. To determine whether this is the case, we compared the gene content of the phage genomes. Orthologous proteins between two phage genomes were identified by reciprocal blast. We then calculated the similarity of a pair of genomes as the number of orthologs divided by the number of proteins in the smaller genome. The average pairwise similarity of phage genomes is 18%, whereas a pair of CRISPR ^+^ phages has an average similarity of 24%. Thus the results are partly confounded by the relatedness of the phages, but the majority of the genes are different for a pair of genomes.

#### CRISPR type II-A-1

Different PAMs in different species and CRISPR loci were observed for CRISPR type II-A. We again identified the motif bioinformatically (Additional file 1: Figure S2): for type II-A-1, we found the PAM GGNG for *S. thermophilus* and GG for *S. mutans* and for the pyogenic group. GG is the motif or a submotif of all the PAMs observed for type II-A-1. It is generally underrepresented in the studied phages, and there is no evidence of a difference between the two groups (Table 4).

**Table 4:** **Summary of the statistical results using the Wilcoxon rank-sum test for CRISPR type II and the extended Streptococcus data set**

		***r***_PAM_				
Type	PAM	CRISPR ^-^	CRISPR ^+^	Difference	***p*** -value	Method
II-A-1	GG	-0.1531	-0.1903	0.03716	0.1238	res
II-A-1	GG	-0.1134	-0.1074	-0.005987	0.9595	sub
II-A-1	GGNG	-0.1332	-0.1872	0.05395	0.1441	res
II-A-1	GGNG	0.1413	0.1160	0.02531	0.6112	sub
II-A-1*	GGNG	-0.1438	-0.1995	0.05579	0.02117	res
II-A-1*	GGNG	0.1513	0.05722	0.09411	0.002451	sub
II-A-2*	AGAAW	0.06871	0.01334	0.05537	0.2901	res
II-A-2*	AGAAW	0.02110	-0.06412	0.08522	4.983e-06	sub
II-A-2*	AAAAW	-0.03673	-0.1521	0.1212	3.18e-05	res
II-A-2*	AAAAW	0.002862	0.03048	-0.02762	0.131	sub
II-A-2*	ANAAW	-0.06373	00.1625	0.09880	1.125e-07	res
II-A-2*	ANAAW	-0.01082	-0.005876	-0.004943	0.8050	sub

See also caption in Table 2. * - only *S. thermophilus* phages in CRISPR^+^, the other phages in CRISPR^-^. The column “Strain resampling” is omitted here since there are no significant results for the first four lines and the test is not applicable to the other lines.

**Figure 6:**
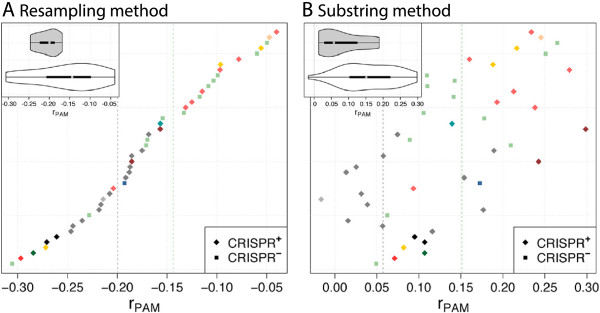
***r***_**PAM**_ **-values for type II-A-2, PAM GGNG, and genus** ***Streptococcus*** **.** Main plot: Median of *r*_PAM_ for *S. thermophilus* is given as a gray line and for all other species as a green line. Inserted figure: gray - *S. thermophilus*, white - other species. In addition, see legend in Figure 5. Phages are ordered by *r*_PAM_ of the resampling method (bottom to top): 23782, phi3396, PH10, SMP, M102, M102AD, 5093, Sfi21, 7201, 11865, Sfi11, 2972, YMC-2011, 858, 315.1, PH15, TP-J34, Abc2, DT1, LYGO9, Sfi19, O1205, EJ-1, 8140, ALQ13.2, JX01, SM1, Dp-1, 34117, 315.5, 315.4, 040922, 315.3, Cp-1, V22, 2167, 315.2, phiNJ2, phi-m46.1, MM1_1998, phi-SsUD.1, MM1, P9, 315.6.

The motif GGNG has only been described to be the PAM for *S. thermophilus* and not for other *Streptococcus* phages. As expected, it is underrepresented in *S. thermophilus* phages (dark gray in Figure 6), but not in most other CRISPR ^+^ phages. Indeed, when including only the *S. thermophilus* phages in the CRISPR ^+^ group, the results were significant. The analysis further indicates that *S. salivarius* might share this longer motif as well (light gray in Figure 6). Note that the difference between groups of phages is largely consistent between the two methods, but the absolute log-ratios are not. Using the resampling method, the motif GGNG seems generally underrepresented, whereas using the substring method the motif seems generally overrepresented. The latter can be explained by the expected frequencies being based on G-rich substrings that are themselves underrepresented in these species. Notably, apart from the deviation in the absolute value, both methods detected the difference between *S. thermophilus* phages and other phages.

#### CRISPR type II-A-2

Type II-A-2 known as CRISPR1 in *S. thermophilus*, has the well-described acquisition motif AGAAW [22]. Cas9 has recently been shown to use the interference motif AAAAW *in vitro*[24]. Here, we found the PAM ANAAW computationally for *S. thermophilus*. The sequence differs from the PAM found for the mitis group, which is AAAG based on eight protospacers (Additional file 1: Figure S2). It is not analyzed here due to the small number of observations and the lack of additional evidence. All different PAM versions show no consistent pattern of underrepresentation in *S. thermophilus* phages (Table 4, Additional file 1: Figure S4). The acquisition motif AGAAW shows a significant underrepresentation only with the substring method. This result can also be obtained when the strands are considered separately or the submotifs AGAAA or AGAAT are analyzed (Additional file 1: Figure S5).

## Discussion

Here, we tested the hypothesis that selection favors the depletion of PAMs in genomes of phages with CRISPR containing hosts, but not in phages infecting hosts without the CRISPR locus. We found support for this hypothesis in some, but not all, of the genera and CRISPR systems tested.

In detail, the PAM GAA for type I-C is generally underrepresented in phages infecting bacterial species having CRISPR (CRISPR ^+^) compared to phages infecting bacterial species not having CRISPR (CRISPR ^-^). This is consistent with the observation that this PAM was found in all the systems studied (Table 1). We analyzed all bacterial genera with at least three CRISPR ^+^ and at least three CRISPR ^-^ phages. Of three genera fulfilling these conditions, the depletion is particularly strong in the *Streptococcus* phages. PAMs for type I-E are variable and, thus, not generally depleted in CRISPR ^+^. In Gammaproteobacteria, the PAMs AAG and AWG were found. No conclusive pattern of depletion was found in Gammaproteobacteria, as only one of the two methods indicates underrepresentation of these two motifs. Of two genera that can be analyzed in detail, *Vibrio* shows a clear depletion of both PAMs in CRISPR ^+^ compared to CRISPR ^-^. Note that the absolute ratios are around 0 or even larger than 0 for most *V. cholera* phages. Thus, the depletion can only be recognized through a comparison with other *Vibrio* phages. In bacteria other than proteobacteria, the PAM AAY was found. No evidence of depletion was found, but this motif might not generalize to the species studied here. PAMs for type II were mainly studied for *Streptococcus*. There is evidence for an underrepresentation of the PAM GGNG in *S. thermophilus*. Taken together, the analysis based on species of one genus allows the identification of particular bacteria species with phages under selection for depletion of PAMs. In most analyses, we combine alternative motifs into one consensus motif and also ignore any strand bias. Although a priming mechanism might lead to the acquisition of further spacers on a strand with an existing spacer [32], there is no known bias for the first spacer. We found concordant results for the depletion measures for both strands.

Several groups gave inconclusive results where only one method indicated underrepresentation in CRISPR ^+^. For CRISPR1 in *S. thermophilus*, the acquisition motif AGAAW is well studied. Although the ratios for this motif are smaller in CRISPR ^+^ compared to CRISPR ^-^ for both methods, the difference is significant only for the substring method. Our results contradict previous conclusions for *Streptococcus thermophilus*[15]. Savitskaya et al. found no significant underrepresentation of AGAAW using a z-score that is also based on substrings. Two main methodological differences might explain this discrepancy. First, we did not assume the pressence of an *a priori* threshold, but compared the statistic to phages infecting other species of the same genus not having a CRISPR system that utilizes that particular PAM. In contrast, a conservative z-score threshold of -3 was used by Savitskaya et al. Second, we subsumed the counts for all four motifs (AGAAA, AGAAT, and the reverse complements) into one ratio, whereas Savitskaya et al. tested each motif separately and required that these separate analyses led to significant results. If we analyze the strands separately or the submotifs separately we also find a significant underepresentation with the substring method (Additional file 1: Figure S5). The z-scores presented by Savitskaya et al. for *S. thermophilus* clearly tend to be negative rather than positive, indicating underrepresentation. We thus conclude that our method that subsumes the frequencies for alternative motifs into one statistic and compares it with phages infecting other species is more powerful.

Our evidence for PAM depletion in some phage species adds to a growing literature on the way evolutionary forces shape the oligonucleotide content of microbial genomes. Genomic sequences carry species-specific signals termed genomic signatures [26, 33]. These signatures are useful for the classification of metagnomic sequences [34, 35]. Local divergences in genomic signatures carry signals for the detection of genomic islands and horizontally transferred genes [36, 37]. Similarities in signatures can be used to compute distance-based phylogenies for bacteria [38] or viruses [39, 40], and they also support a co-evolution in signatures between bacteriophages and hosts [26, 40]. Frequencies of certain motifs have been used to study selection acting on bacteria and phage genomes. CpG nucleotides are underrepresented in some eukaryotic viruses [41], and this could be due to the mimicking of the host composition to avoid immune recognition [42]. Bacterial genomes are also depleted from spurious transcription factor binding sites due to weak selection [43]. Furthermore, palindromes are underrepresented in phage and bacterial genomes due to restriction site avoidance [27, 44].

Note that the role of CRISPR as a bacterial immune system has been questioned [45]. To our knowledge, only in *Streptococcus* species, natural bacterial strains show CRISPR expansion after phage challenge, thus the role of the system in other species is even more obscure. In *Escherichia coli*, CRISPR expansion after phage challenge was only observed when overexpressing the cas genes [32]. In concordance with this, no PAM avoidance was observed in *Escherichia coli* phages [15]. Here, the ratios for the PAM AAG do also not tend to be negative for 93 *E. coli* phages (see Additional file 2). Note, however, that the approach of comparing the ratios to closely related CRISPR ^-^ phages is more powerful for *Vibrio* (Figure 4), which shows positive ratios, but lower ratios are observed in CRISPR ^+^. However, this approach is not possible for *E. coli*. In addition, the acquisition motif for *E. coli* was recently shown to comprise more positions than the PAM [46]. This might have resulted in a weaker selection pressure on individual positions of the PAM than previously thought.

The dynamic nature of the system complicates the analyses presented in some cases. CRISPR/Cas loci are often horizontally transferred or inactivated (e.g., [47, 48]). In addition, changes in the PAM sequence occur during evolution. Thus, the evolutionary history affects the selection pressure on the phages over time and has an influence on what is detectable today. Our observation of a depletion despite the fact that CRISPR/Cas systems are dynamic and often occur in labile genomic regions, is surprising, because an ongoing selection pressure is needed for this observation. However, note that CRISPR is not only dynamic between bacterial species but in particular also within species (see e.g., Additional file 1: Table S1) and CRISPR systems were postulated to be in a constant flux as a result of trade-off between positive and negative selection [48]. Thus, we hypothesize that, in bacterial populations, CRISPR is dynamic but is maintained long-term in at least some strains of a species which can result in a detectable selection pressure on phages.

In addition to the dynamic nature of the CRISPR locus, other factors might have influenced the phenomenon where only phages infecting some groups of bacteria show a depletion in PAMs, while other phages do not. Expansion of the CRISPR locus after phage challenge was only observed for some bacterial species. In other species, other defense systems may have played more important roles or bacteria could have evolved resistance more effectively by other means, for example, with mutations that prevented phage adsorption. In this case, phages that we annotated as CRISPR ^+^ may not encounter the CRISPR system. Another factor that has an impact on the analyses are the phage-host relationships. The host annotation could not include the full host range of the phage and the annotated host may not even be a typical or frequent host for that phage.

In the bacterial species where the CRISPR system has an important role in bacteriophage infection, selection pressure on PAMs may have been high. Note that at least in type I-E, the PAM is thought to be required for the initial recognition and dsDNA helix destabilisation [49]. Furthermore many more phage mutations escaping CRISPR recognition occur in the PAM compared to in the protospacer sequence (e.g., in [45] of 15 escape mutants, two had mutations only in the protospacer region, twelve only in the PAM and one in both). This is also an indication that selection pressure on PAMs is high.

## Conclusions

The analyses presented here compare phages infecting hosts with and without CRISPR and, thus, have the power to show that phage genomes are under selection due to targeting by CRISPR systems. We observe a depletion of PAMs in phage genomes infecting hosts harboring CRISPR systems in some groups of bacteria. Thus, there is a selection pressure against PAMs even in the presence of dynamic CRISPR/Cas systems. This indicates that the systems have been acting in an efficient way in the species discussed here.

The results presented here might be driven by only some hosts, and detailed analyses based on genera could only be done for few genera. In the future, with more phage genomes and more bacteria with CRISPR information available, it will be possible to systematically test the selection hypothesis across a more diverse assemblage of species.

Besides phage genome evolution, the CRISPR system might additionally have an effect on the evolution of other microbial parasites, like plasmids and other mobile elements. In future work, it would be interesting to investigate wether the CRISPR system also has an impact on plasmid evolution and to investigate its relative impact on phage and plasmid genomes.

## Methods

### Phage data set for all bacteria

We downloaded the list of sequenced bacteriophages from NCBI (http://www.ncbi.nlm.nih.gov/genomes/Geno mesGroup.cgi?opt=virus&taxid=10239&host=bacteria). We retained all genomes with the following criteria: (i) annotated CDS, (ii) annotated host with the /host-tag in the genbank file and (iii) at least one complete genome for the host species in RefSeq v5.8. This resulted in 588 phage genomes. The host specificity of phages was assigned according to the /host-tag. Cas genes in the bacterial genomes were annotated using hmmsearch on the RefSeq database [50] and pre-defined Pfam alignments [2] for types I-C and I-E. The type was still assigned if some cas genes are absent but at least one of the subgroup-specific genes (*csd* for type I-C and *cse* for type I-E) is present.

PAM sequences are thought not to be required for type III [2], thus we focus on types I and II. Known PAM sequences for these types are summarized in Table 1. Some subtypes of types I and II are well studied and are represented in current data sets. Thus, we only focus on some subtypes and do not specifically analyze other subtypes. The PAM GG occurrs for multiple CRISPR types, namely I-A, I-B, I-F, and II. This short motif contains little information and was excluded from most analyses. We also do not consider type I-B motifs as this type occurs mainly in Archaea and seems to recognize a larger number of PAM sequences. For type I-C, the PAM GAA has been found in different species with computational methods. Type I-D is only studied in one species, thus, there is not enough evidence to show whether this motif might apply to other species. Type I-E is extensively studied in *E. coli*, where it shows the PAM AWG. AAG is the acquisition motif and the interference motif is broader. Notably, this motif was only found in *E. coli*, *Erwinia amylovora*, and *Pseudomonas aeruginosa* and might apply only to Gammaproteobacteria. For other species, the PAM AAY has been found using computational methods. The motifs for type II seem variable and are mainly studied in *Streptococcus*. Taken together, this information shows that type I-C seems to have a constant PAM and is a suitable type to study motif underrepresentation across different bacterial species. The motif for type I-E is variable across the phylogeny, but underrepresentation for some groups can be assessed. Type II has mainly been studied in *Streptococcus*, thus we limit our analysis for type II to this genus.

### Streptococcus data set

We downloaded all available complete genomes of *Streptococcus* and all phages infecting that group from NCBI. This resulted in 98 complete bacterial genomes and 43 contig-state bacterial genomes (Additional file 1: Tables S1,S2). Contig-state genomes were only included from species with at least one phage genome infecting that species excluding *Streptococcus pneumoniae*. The latter was ignored since no cas genes were found among the 24 complete genomes and thus contig-state genomes were not expected to yield further information. There were 44 phage genomes with an annotated host species (Additional file 1: Table S3). Note that this data set comprises phages not present in the first data set. First, some were not listed in NCBI genomes. Second, they may not had a /host tag in the genbank file, but the name clearly indicates the host species.

Orthologs were determined for the 98 complete bacterial genomes. Best bidirectional blastp hits determined pairwise orthologs. An orthologous group was required to comprise one protein from each genome and all proteins had to be pairwise orthologs with all other proteins in the group. This conservative approach gave rise to 424 orthologous groups, this is less than the previously described *Streptococcus* core genome size of 600 [51]. They were aligned with MAFFT using the auto option [52], alignment columns were masked using ZORRO with a confidence score cutoff of 5 [53]. This results in an alignment of 131,439 sites. Phylogenies were calculated with PHYML under the LG model [54].

Cas genes were annotated using HMMer [55] and pre-defined Pfam alignments [2] for all bacterial genomes and contigs. CRISPR/Cas types were assigned according to the proposed scheme [2]. The array of cas genes resulting in a defined CRISPR/Cas type is referred to as the *cas locus*. A type is still assigned if some cas genes are absent but the type is identifiable. Overall, 101 cas loci were found.

CRISPRs were detected computationally using CRISPR finder [56] on all bacteria genomes and on contigs where a cas locus was found in the previous step. CRISPRs were assigned to the most proximal cas locus, in a few cases the repeat detected by CRISPRfinder was changed manually to match homologous CRISPR repeats. Overall, 89 CRISPR arrays were found; three of them could not be matched to a cas locus, one cas locus had two CRISPR arrays and the other matches were unambiguous (Additional file 1: Table S2).

Protospacers were detected with needleall from EMBOSS v6.3.1 [57] and 80% sequence identity over the length of the spacer using the spacers identified in the previous step. Here, protospacer denotes a sequence in the phage genome with a similar sequence as a spacer from a CRISPR array. That means, we ignore which strand actually binds to the target and do not consider the reverse complement. Note that this definition differs from previous definitions [31].

Prophages were annotated with PHAST [58] for complete genomes.

### Underrepresentation measures

#### Resampling method

Here we applied the method described by Robins et al. [26]. A motif *m*=*m*_1_…*m*_*n*_ of length *n* is called an *n*-string. Its frequency is counted in all coding regions on positions that span codon boundaries. In detail, for *n*>3, this is simply the frequency of *m* in coding regions; for *n*=3, it is the frequency among all 3-strings starting on the second or third codon position; and for *n*=2, it is the frequency among all 2-strings starting at the third codon position. This results in the frequency *N*_*m*_. Then *s* resampled genomes are generated. For each resampled genome, each open reading frame is resampled independently, thereby all synonymous codons inside one open reading frame are reshuffled randomly. This method generates new codon boundaries and an expected measure of the motif frequency  for iteration *i*. The ratio of observed over expected frequencies is given by .

#### Substring method

This method has been called Markov method when applied to a fixed substring length [27], but here, the substring length is determined by the motif length *n*. The observed frequency is simply the frequency of *m* in the genome, *f*(*m*). The expected frequency is given by , then .

#### Test statistic

These measures of underrepresentation were applied to each phage genome independently to yield a ratio for each genome and method. Then, the genomes were divided in two sets, one where the host species has CRISPR (CRISPR ^+^) and one where it did not have CRISPR (CRISPR ^-^). The difference in *r*_PAM_-values between CRISPR ^+^ and CRISPR ^-^ was analyzed using a Wilcoxon rank-sum test.

We use a resampling with replacement method to generate data sets with a uniform host species distribution (“Strain resampling”). For each sample, a data set of the same size as the original one was generated. For each element of the sample, a species was first chosen randomly with each species being equally likely, then a strain of that host genome was chosen uniformly.

Plotting and statistical analysis were done with R [59].

## Electronic supplementary material

Additional file 1: **Table S1** - Summary of the *Streptococcus* data set. **Table S2** - *Streptococcus* data for hosts. **Table S3** - *Streptococcus* phage genomes grouped by host. **Figure S1** - *Streptococcus* phylogeny. **Figure S2** - Logo of positions adjacent to protospacers for *Streptococcus* data set. **Figure S3** - Distributions of *r*_PAM_ for type I-C and genus *Streptococcus* using prophages. **Figure S4** - Distributions of *r*_PAM_ for type II-A-2 and two different measures of underrepresentation. **Figure S5** - Submotifs of the motif AGAAW (type II-A-2). (PDF 269 KB)

Additional file 2: **Hosts sheet** - Information for bacterial species with sequenced genomes and sequenced phage genomes. For each bacterial species, information about absence (0) or presence (1) in at least one strain of this species is given for CRISPR types I-C and I-E. **Ratios sheet** - *r*_PAM_ values for the motifs analyzed for all bacteria. Motifs on both strands were subsumed into one ratio. (XLSX 167 KB)

Additional file 3: ***r***_**PAM**_ **values for the motifs analyzed in the text for the** ***Streptococcus*** **data set.** Motifs on both strands were subsumed into one ratio. (XLSX 53 KB)

## Abbreviations

Cas gene: CRISPR associated gene
CRISPR: Clustered regularly interspaced short palindromic repeats
CRISPR^+^: Phages associated with hosts harboring CRISPR/Cas
CRISPR^-^: Phages associated with hosts not harboring CRISPR/Cas
crRNA: CRISPR RNA
PAM: Protospacer associated motif.
