# Supplementary material for: Motif depletion in bacteriophages infecting hosts with CRISPR systems
Source: BMC Genomics. 2014 Aug 8;15(1):663. doi: 10.1186/1471-2164-15-663 (PMC4246573; doi:10.1186/1471-2164-15-663)
Supplement: Supplementary file 1 — Additional file 1: Table S1 - Summary of the Streptococcus data set. Table S2 - Streptococcus data for hosts. Table S3 - Streptococcus phage genomes grouped by host. Figure S1 - Streptococcus phylogeny. Figure S2 - Logo of positions adjacent to protospacers for Streptococcus data set. Figure S3 - Distributions of r PAM for type I-C and genus Streptococcus using prophages. Figure S4 - Distributions of r PAM for type II-A-2 and two different measures of underrepresentation. Figure S5 - Submotifs of the motif AGAAW (type II-A-2). (PDF 269 KB) [file 12864_2014_6677_MOESM1_ESM.pdf]

Table S1: Summary of the *Streptococcus* data set. Contig-state genomes for *S. pneumoniae* and for species without known phages were not looked up (marked "-"). Prophages were annotated with PHAST for complete genomes. A CRISPR/Cas type is reported if a defined array of cas genes is detected. They are listed for complete and contig-state genomes together. A detailed list of bacteria and phage genomes used can be found in Tables S2 and S3.

	Genomes				CRISPR/Cas				
Species	Complete	Contig-state	Phages	Prophages	I-C	I-E	I-F	II-A-1	II-A-2
Salivarius group									
S. thermophilus	6	3	12	3	0	0	0	3	9
S. salivarius	3	4	1	3	0	3	0	1	2
S. mutans	4	0	2	1	3	2	0	4	0
Bovis group									
S. infantarius	1	-	0	1	0	0	0	1	0
S. macedonicus	1	-	0	4	0	0	0	0	1
S. pasteurianus	1	-	0	1	0	0	0	0	1
S. gallolyticus	3	-	0	4	0	0	0	3	2
Pyogenic group									
S. agalactiae	5	9	2	8	2	0	1	14	0
S. uberis	1	-	0	1	0	0	0	0	0
S. parauberis	1	-	0	3	0	0	0	0	0
S. equi	4	0	1	5	3	0	0	1	0
S. dysgalactiae	5	2	1	24	4	0	0	5	0
S. pyogenes	17	1	7	70	9	0	0	13	0
S. suis	14	2	3	18	0	0	0	1	3
S. intermedius	1	-	0	1	0	0	0	0	0
Mitis group									
S. parasanguinis	2	-	0	0	1	0	0	0	0
S. sanguinis	1	-	0	0	0	0	0	0	0
S. gordonii	1	0	1	0	0	0	0	0	1
S. oralis	1	9	1	1	0	1	0	3	2
S. mitis	1	13	1	3	0	0	0	1	1
S. pseudopneumoniae	1	-	0	4	0	0	0	0	0
S. pneumoniae	24	-	12	39	0	0	0	0	0
Total	98	43	44	194	22	6	1	50	22

Table S2: *Streptococcus* data for hosts. Number of contigs is given for contig-state genomes. For strains with a cas locus, the number of spacers is given or 'x' if Cas but no CRISPR was detected.

				CRISPR/Cas				
Accession	Strain	Contigs	Prophages	I-C	I-E	I-F	II-A-1	II-A-2
Streptococcus agalactiae								
AE009948	2603V/R	-	3				24	
AL732656	NEM316	-	0				13	
CP000114	A909	-	3			x	14	
CP003810	GD201008-001	-	1				8	
CP003919	SA20-06	-	1	x			x	
AAJO01000000	18RS21	553	-				x	
AAJP01000000	515	255	-				12	
AAJQ01000000	CJB111	155	-				17	
AAJS01000000	H36B	345	-				13	
AEQQ01000000	ATCC 13813	134	-				8	
AEXT01000000	FSL S3-026	8	-				14	
AKAP01000000	ZQ0910	54	-				1	
AKXO01000000	GB00112	56	-				9	
ALXB01000000	STIR-CD-17	96	-	x			x	
Streptococcus dysgalactiae								
AP010935	GGS_124	-	5				18	
AP011114	RE378	-	4	13			7	
CM001076	ATCC 27957	-	7	1				
CP002215	ATCC 12394	-	1	29			25	
HE858529	AC-2713	-	7	14			19	
AFIN01000000	SK1249	231	-				11	
AFUL01000000	SK1250	1	-					
Streptococcus equi								
CP001129	MGCS10565	-	0	9			17	
CP002904	ATCC 35246	-	1	18				
FM204883	4047	-	4					
FM204884	H70	-	0	18				
Streptococcus gallolyticus								
AP012053	ATCC 43143	-	3				29	10
FN597254	UCN34	-	1				12	15
FR824043	ATCC BAA-2069	-	0				12/15	
Streptococcus gordonii								
CP000725	Challis substr. CH1	-	0					26
Streptococcus infantarius								
CP003295	CJ18	-	1				10	
Streptococcus intermedius								
AP010969	JTH08	-	1					
Streptococcus macedonicus								
HE613569	ACA-DC 198	-	1					50

Continued on next page

Table S2: *Streptococcus* data for hosts. Number of contigs is given for contig-state genomes. For strains with a cas locus, the number of spacers is given or 'x' if Cas but no CRISPR was detected.

				CRISPR/Cas				
Accession	Strain	Contigs	Prophages	I-C	I-E	I-F	II-A-1	II-A-2
Streptococcus mitis								
FN568063	B6	-	3					
AEDU01000000	SK564	35	-					
AEDT01000000	SK321	40	-				5	
AEDV01000000	SK597	108	-					
AEDX01000000	NCTC 12261	24	-					
AEEN01000000	ATCC 6249	20	-					47
AFQT01000000	SK1073	54	-					
AFUB01000000	SK95	66	-					
AFUF01000000	SK569	54	-					
AFUO01000000	F0392	2	-					
AICR01000000	SK616	126	-					
AICU00000000	SK575	90	-					
AJJL00000000	SK579	82	-					
ALCH00000000	SPAR10	10	-					
Streptococcus mutans								
AE014133	UA159	-	1	x			5	
AP010655	NN2025	-	0		18		69	
AP012336	LJ23	-	0	14			8	
CP003686	GS-5	-	0	1	20		21	
Streptococcus oralis								
FR720602	Uo5	-	1					
ADMV01000000	ATCC 35037	28	-					
AEDW01000000	ATCC 35037	25	-					
AFNM01000000	SK255	63	-				16	
AFUU01000000	SK313	5	-					41
AICT00000000	SK1074	20	-					4
AJKO00000000	SK10	39	-		8			
AJKP00000000	SK100	51	-					
AJKQ00000000	SK610	31	-				31	
ALJN00000000	SK304	25	-				31	
Streptococcus pasteurianus								
AP012054	ATCC 43144	-	1					37

Continued on next page

Table S2: *Streptococcus* data for hosts. Number of contigs is given for contig-state genomes. For strains with a cas locus, the number of spacers is given or 'x' if Cas but no CRISPR was detected.

				CRISPR/Cas				
Accession	Strain	Contigs	Prophages	I-C	I-E	I-F	II-A-1	II-A-2
Streptococcus pneumoniae								
AE005672	TIGR4	-	1					
AE007317	R6	-	1					
CP000410	D39	-	0					
CP000918	70585	-	1					
CP000919	JJa	-	2					
CP000920	P1031	-	1					
CP000921	Taiwan19F-14	-	1					
CP000936	Hungary19A-6	-	1					
CP001015	G54	-	1					
CP001033	CGSP14	-	1					
CP001845	gamPNI0373	-	2					
CP001993	TCH8431/19A	-	2					
CP002121	AP200	-	2					
CP002176	670-6B	-	4					
CP003357	ST556	-	4					
FM211187	ATCC 700669	-	1					
FQ312027	OXC141	-	2					
FQ312029	INV200	-	3					
FQ312030	INV104	-	2					
FQ312041	SPN994038	-	1					
FQ312043	SPN034183	-	1					
FQ312044	SPN994039	-	1					
FQ312045	SPN034156	-	1					
HE983624	SPNA45	-	3					
Streptococcus pseudopneumoniae								
CP002925	IS7493	-	4					
Streptococcus parasanguinis								
CP002843	ATCC 15912	-	0	19				
CP003122	FW213	-	0					
Streptococcus parauberis								
CP002471	KCTC 11537	-	3					

Continued on next page

Table S2: *Streptococcus* data for hosts. Number of contigs is given for contig-state genomes. For strains with a cas locus, the number of spacers is given or 'x' if Cas but no CRISPR was detected.

				CRISPR/Cas				
Accession	Strain	Contigs	Prophages	I-C	I-E	I-F	II-A-1	II-A-2
Streptococcus pyogenes								
AE004092	M1 GAS	-	3	3			6	
AE009949	MGAS8232	-	5					
AE014074	MGAS315	-	7				x	
AM295007	Manfredo	-	5					
BA000034	SSI-1	-	6				x	
CP000003	MGAS10394	-	7					
CP000017	MGAS5005	-	4	4			3	
CP000056	MGAS6180	-	3	1			4	
CP000259	MGAS9429	-	4	7			x	
CP000260	MGAS10270	-	4	3			x	
CP000261	MGAS2096	-	3		6		x	
CP000262	MGAS10750	-	4	5			x	
CP000829	NZ131	-	4	5			4	
CP003068	Alab49	-	5					
CP003116	MGAS15252	-	1				2	
CP003121	MGAS1882	-	1				3	
CP003901	A20	-	4	4			3	
CAHN01000000	NS88.2	298	-					
Streptococcus salivarius								
CP002888	57.I	-	1		x			
FR873481	CCHSS3	-	1		x			
FR873482	JIM8777	-	1					x
ACLO01000000	SK126	101	-					
AGBV01000000	M18	6	-					
AJFW01000000	PS4	56	-		2			2
ALIF01000000	K12	7	-				15	
Streptococcus sanguinis								
CP000387	SK36	-	0					
Streptococcus suis								
AM946016	P1/7	-	0					
CP000407	05ZYH33	-	2					
CP000408	98HAH33	-	0					
CP000837	GZ1	-	0					
CP002465	JS14	-	3					
CP002570	A7	-	0					
CP002633	ST3	-	3					49
CP002640	SS12	-	2					
CP002641	D9	-	1					x
CP002644	D12	-	3					
CP002651	ST1	-	4				x	
CP003736	S735	-	0					
FM252031	SC84	-	0					
FM252032	BM407	-	0					
AAFA03000000	89/1591	82	-					6
AEYY01000000	R61	53	-					

Continued on next page

Table S2: *Streptococcus* data for hosts. Number of contigs is given for contig-state genomes. For strains with a cas locus, the number of spacers is given or 'x' if Cas but no CRISPR was detected.

				CRISPR/Cas				
Accession	Strain	Contigs	Prophages	I-C	I-E	I-F	II-A-1	II-A-2
Streptococcus thermophilus								
CP000023	LMG 18311	-	0					33
CP000024	CNRZ1066	-	1					41
CP000419	LMD-9	-	1				8	16
CP002340	ND03	-	0				20	36
CP003499	MN-ZLW-002	-	1				26	30
FR875178	JIM 8232	-	0					42
AGFN01000000	CNCM I-1630	376	-					12
ALIK00000000	MTCC 5460	143	-					x
ALIL01000000	MTCC 5461	144	-					x
Streptococcus uberis								
AM946015	0140J	-	1					

Table S3: *Streptococcus* phage genomes grouped by host. The right column indicates whether the strain is included in the bacteria data set.

Strain	Accession	
Streptococcus agalactiae		
LYGO9	JX409894.1	-
JX01	JX409895.1	-
Streptococcus dysgalactiae		
phi3396	NC_009018.1	+
Streptococcus equi		
P9	NC_009819.1	+
Streptococcus gordonii		
PH15	NC_010945.1	+
Streptococcus mitis		
SM1	NC_004996.1	+
Streptococcus mutans		
M102	NC_012884.1	-
M102AD	DQ386162.1	-
Streptococcus oralis		
PH10	NC_012756.1	+
Streptococcus pneumoniae		
040922	FR671406.1	-
34117	FR671407.1	-
23782	FR671408.1	-
11865	FR671409.1	-
8140	FR671410.1	-
2167	FR671411.1	-
Cp-1	NC_001825.1	+
Dp-1	NC_015274.1	+
EJ-1	NC_005294.1	+
MM1	NC_003050.2	+
MM1_1998	DQ113772.1	-
V22	FR671405.1	-
Streptococcus pyogenes		
phi-m46.1	FM864213.1	-
315.1	NC_004584.1	+
315.2	NC_004585.1	+
315.3	NC_004586.1	+
315.4	NC_004587.1	+
315.5	NC_004588.1	+
315.6	NC_004589.1	+
Streptococcus salivarius		
YMC-2011	NC_018285.1	+
Streptococcus suis		
SMP	NC_008721.2	+
phi-SsUD.1	FN997652.1	-
phiNJ2	JX879087	+

Continued on next page

Table S3: *Streptococcus* phage genomes grouped by host. The right column indicates whether the strain is included in the bacteria data set.

Strain	Accession	
Streptococcus thermophilus		
2972	NC_007019.1	+
5093	NC_012753.1	+
7201	NC_002185.1	+
858	NC_010353.1	+
Abc2	NC_013645.1	+
ALQ13.2	NC_013598.1	+
DT1	NC_002072.2	+
O1205	NC_004303.1	+
Sfi11	NC_002214.1	+
Sfi21	NC_000872.1	+
Sfi19	NC_000871.1	+
TP-J34	NC_020197.1	+

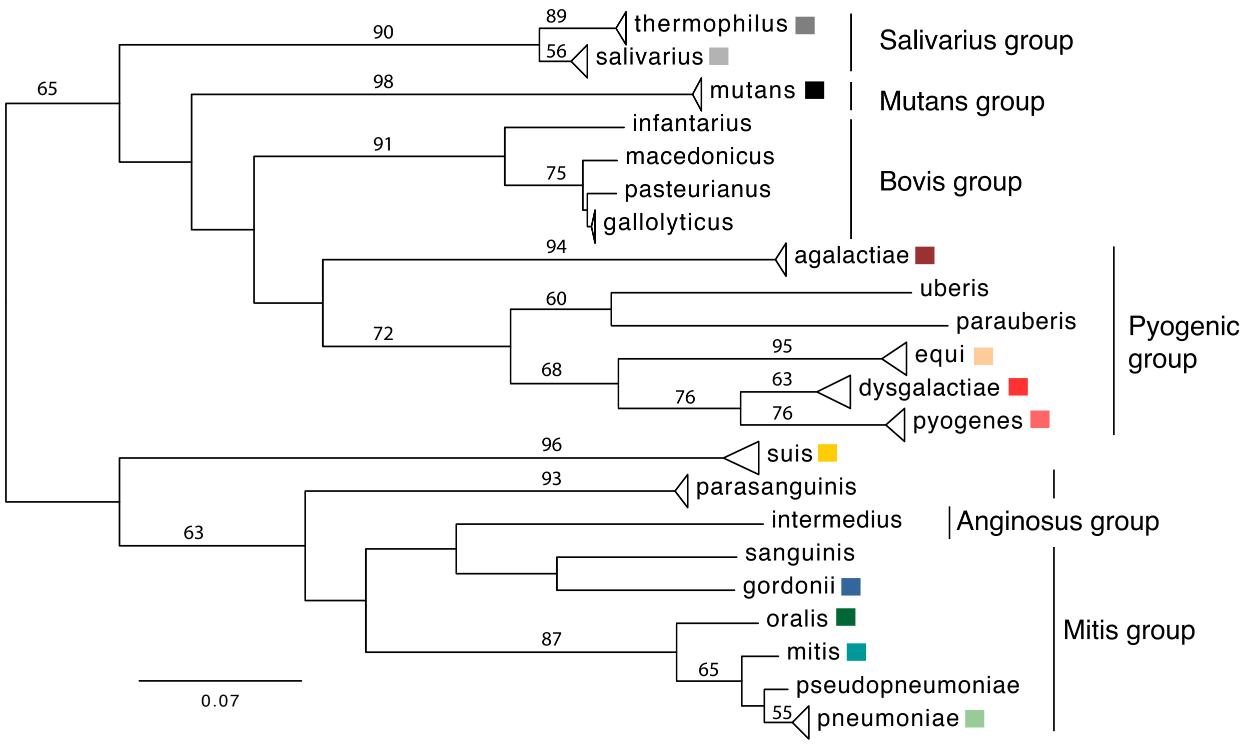

Figure S1: *Streptococcus* phylogeny. Phylogeny of 98 *Streptococcus* species using a superalignment of 424 orthologous proteins with 131,439 sites in total after masking. Percentage of gene trees calculated from individual protein alignments that contain a branch is given for percentages larger than 50. Species are color coded if a phage is known for that species. All trees were computed with PhyML under the LG model. Note that the phylogeny is unrooted.

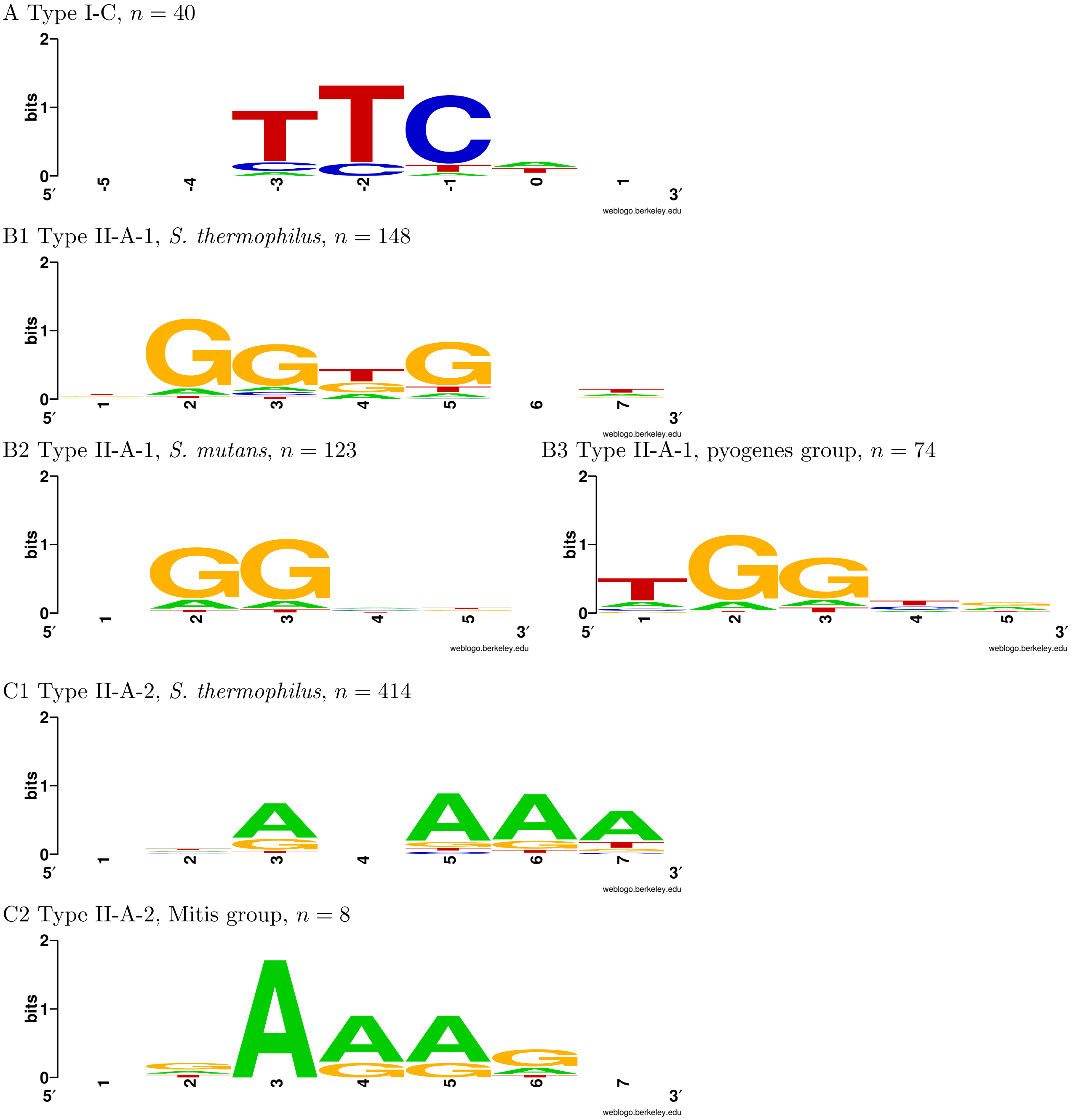

Figure S2: Logo of positions adjacent to protospacers for *Streptococcus* data set. A - protospacer starts at position 0. B,C - 0 is the last position of the protospacer.

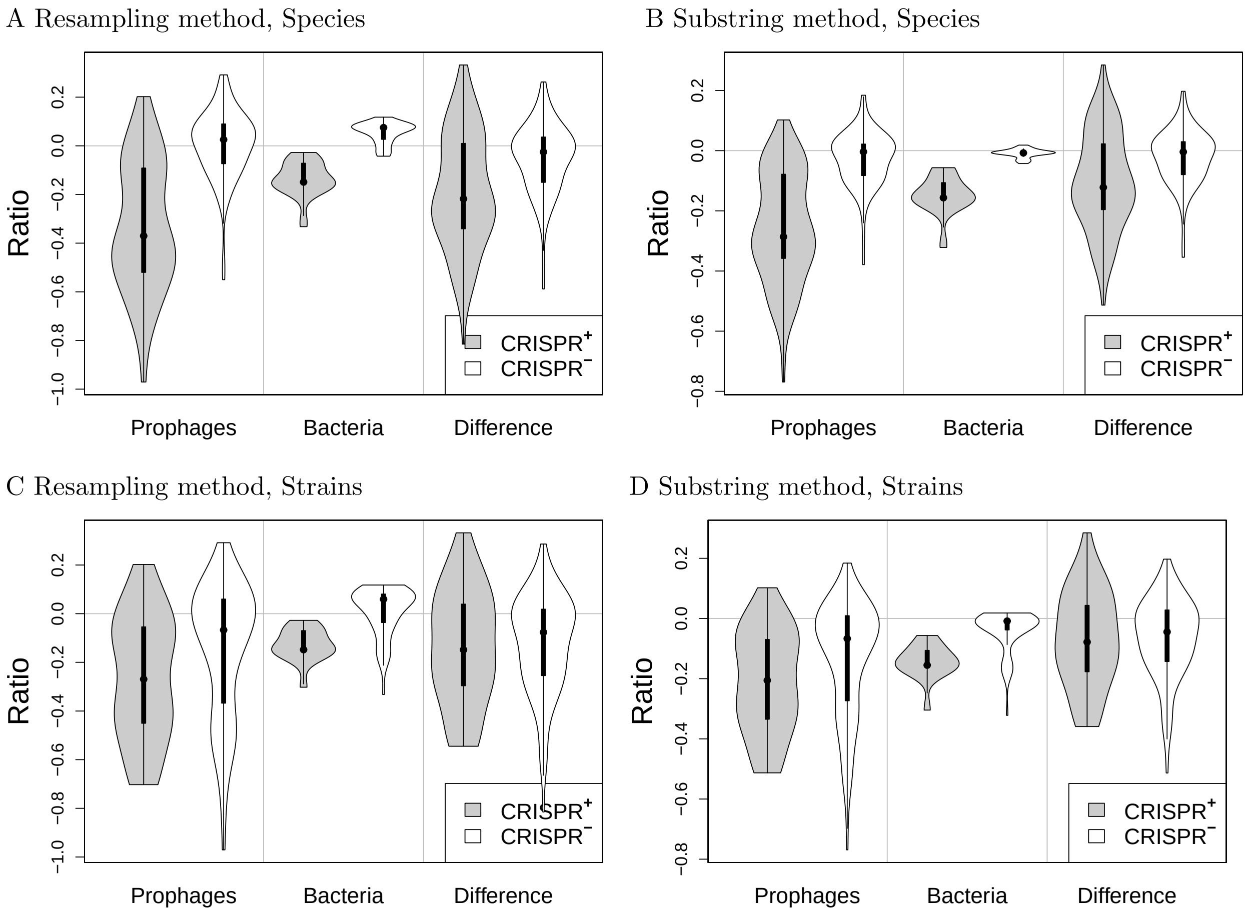

E Summary of the statistical results for the right columns

Difference	p-value	Strain resampling	Method	Note
0.1927	1.203e-05	99	res	Species
0.1181	3.064e-05	98	sub	Species
0.07189	0.5898	n.a.	res	Strains
0.03378	0.6912	n.a.	sub	Strains

Figure S3: Distributions of r_{PAM} for type I-C and genus *Streptococcus* using prophages. A,B All prophages of a species are assigned CRISPR$^+$ if at least one strain of this species carries CRISPR. C,D Only prophages from strains with Cas genes are assigned CRISPR$^+$ independent of the species. The prophages show an underrepresentation if their host species has CRISPR (A,B, left columns). However the bacterial genomes show a similar but weaker pattern (A,B, middle columns). Therefore normalizing the prophage ratio by its host is the basis for an appropriate comparison. The difference of prophage ratios minus host ratios still shows the pattern (A,B, right columns) and is significant (E). If not species but only strains with CRISPR are assigned CRISPR$^+$ (C,D), the difference is not significant. "n.a." - not applicable.

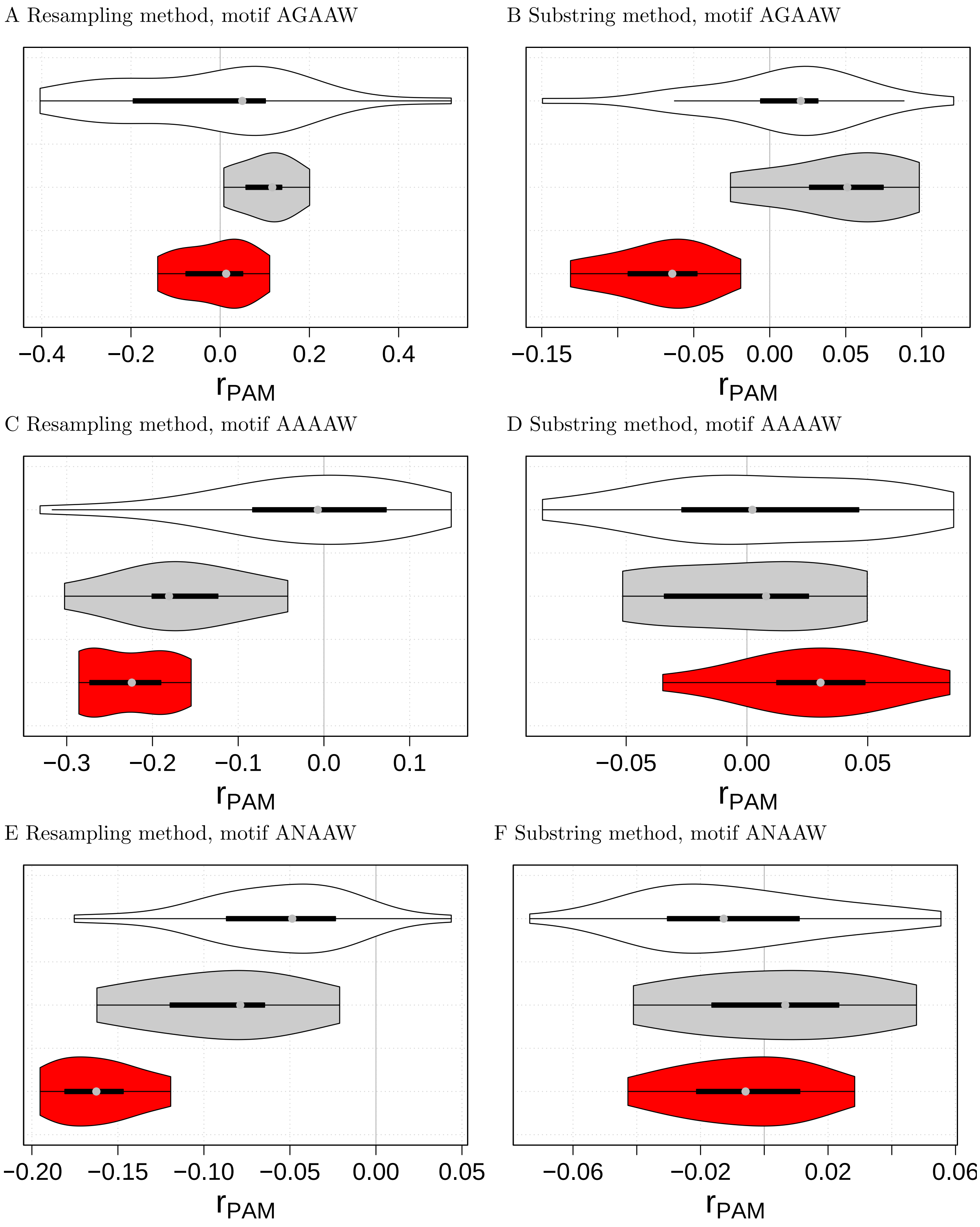

Figure S4: Distributions of r_{PAM} for type II-A-2 and two different measures of underrepresentation. White: CRISPR^{-}, Gray: CRISPR^{+} except *S. thermophilus*, Red: *S. thermophilus*.

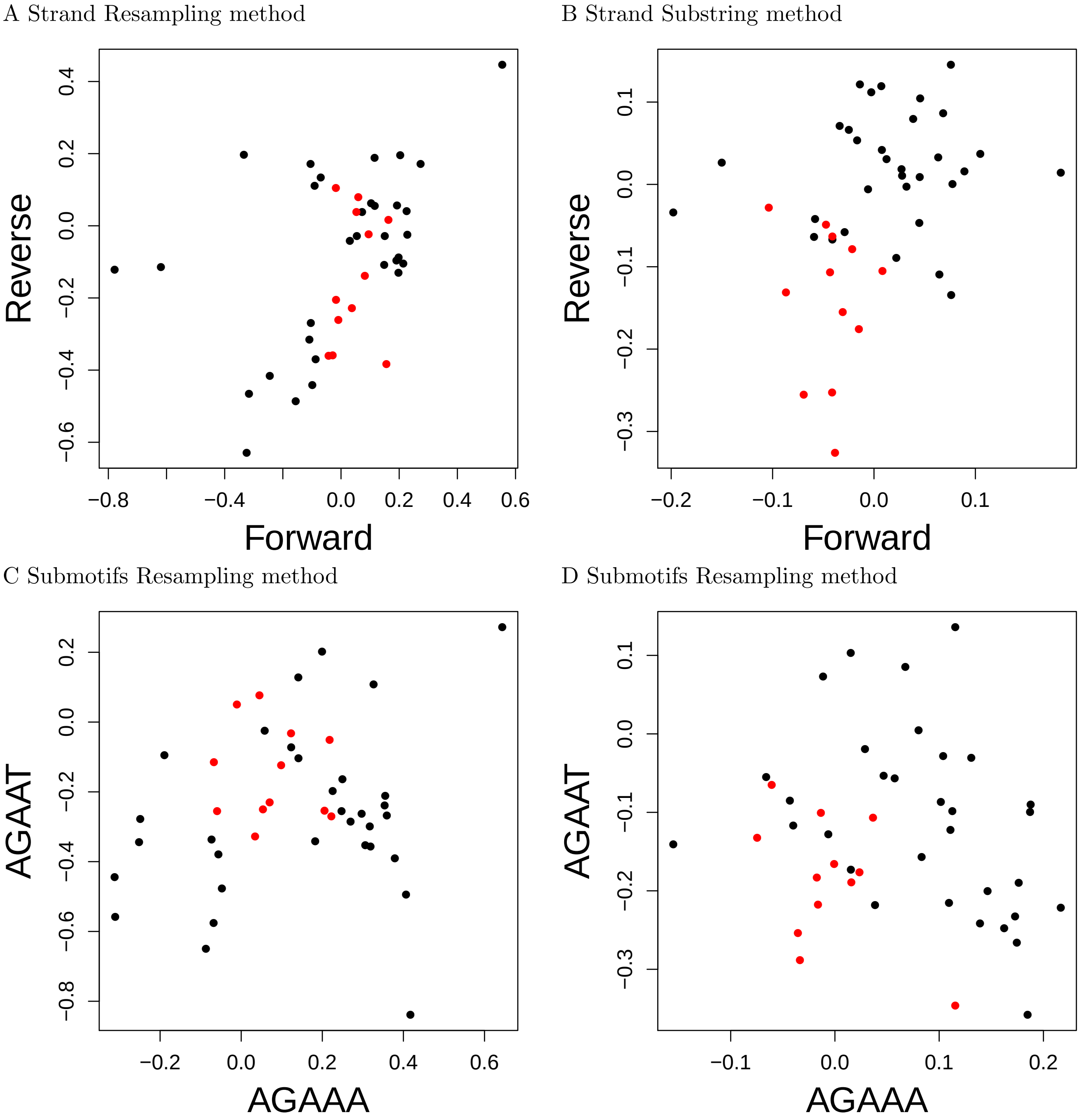

E Summary of the statistical results when only *S. thermophilus* phages are included in CRISPR^+

Motif	Strand	Difference	*p*-value	Method
AGAAW	both	0.05537	0.2901	res
AGAAW	both	0.08522	4.983e-6	sub
AGAAW	forward	0.01810	0.9689	res
AGAAW	forward	0.06578	4.802e-4	sub
AGAAW	reverse	0.1369	0.3277	res
AGAAW	reverse	0.1359	1.518e-6	sub
AGAAA	both	0.01507	0.1528	res
AGAAA	both	0.1176	0.001322	sub
AGAAT	both	-0.1045	0.03995	res
AGAAT	both	0.07167	0.05188	sub

Figure S5: Submotifs of the motif AGAAW (type II-A-2). A,B: Comparison of the different strands. C,D: Comparison of the two submotifs (both strands combined). Red: phages with host *S. thermophilus*, Black: other phages
